# Pediatric Electrophysiology in India: A Sub-speciality Come of Age

**Published:** 2008-05-01

**Authors:** Joy M Thomas, Johnson Francis, KM Cherian

**Affiliations:** 1 Consultant Cardiologist and Chief of Electrophysiology, Frontier Lifeline and Dr KM Cherian Heart Foundation. International Centre for Cardiothoracic and Vascular Diseases, R-30-C, Ambattur Industrial Estate Road, Chennai-600101, India; 2 Professor of Cardiology, Calicut Medical College, Kerala, India; 3 Chairman, Frontier Lifeline and Dr KM Cherian Heart Foundation. International Centre for Cardiothoracic and Vascular Diseases, R-30-C, Ambattur Industrial Estate Road, Chennai-600101, India

**Keywords:** pediatric cardiac electrophysiology, radiofrequency ablation, pacemaker, implantable cardioverter defibrillator

## Introduction

Electrophysiology started in India in the early 70's with the earliest published diagnostic His bundle studies coming from the All India Institute of Medical Sciences by Bhatia ML et al [1] and the GB Pant Hospital by Khalilullah et al [2,3] . That era was remarkable with the first indigenously made temporary pacemaker being used to treat complete heart block as early as in 1970 [4]

## Pacing and Device Therapy in Children

From then on pacemakers have been implanted in all types of cases in every age group and even in difficult situations where the anatomy of the heart defies a via naturalis path for the lead. Epicardial leads were the natural choice in such situations, but the morbidity of the procedure and the high thresholds necessitating frequent pulse generator changes made the electrophysiologist look at alternative routes for the lead [5,6]. The availability of smaller volume (weight) pacemakers have made percutaneous implants feasible in smaller children and the percutaneous route has been regularly used at our institute in children weighing 10 kgs and more.

A day old infant had a pacemaker implanted in an intrathoracic extrapleural location [7]. (Figure 1A) There is a fear that the presence of a pacemaker in the pleural space would prevent the normal expansile growth of the neighbouring lung, but this was belied on followup of the one-day old after two years. (Figure 1B)

Biventricular pacemaker therapy has been applied to the treatment of post-operative children with heart failure, wide QRS and ventricular dys-synchrony [8]. Automatic Implantable Cardioverter Defibrillator devices in children have been implanted in children with long QT syndrome [9] and in post operative children with VT unsuitable for radiofrequency ablation [10] (Figure 2A and B)

## Heritable Arrhythmias in Children

Genetic disorders that cause arrhythmias like the long QT syndrome, Brugada syndrome and arrhythmogenic right ventricular dysplasia have been identified more often now than before due to the greater awareness and the keenness to look for these disorders. Some cases have been treated for years as an epileptic until a chance ECG and an alert pediatrician reveals the actual problem [11].

## Interventional Electrophysiology in Children

After the introduction of Radiofrequency energy for ablation in Humans by Melvin Scheinmann [12,13] there was a flurry of activity all over the world and catheter based ablation for tachyarrhythmias entered India in 1988 when Prof KK Sethi [13] did the first DC ablation and later on used Radiofrequency energy for ablation. Radiofrequency ablation for arrhythmias in children was resorted to only in cases of absolute necessity as in impending tachycardiomyopathy [15-17], (Figure 3A, B and C) in tachyarrhythmias refractory to treatment and in children older than six to seven years of age. In the prevailing socio-economic situation in India where re-use of catheters, duly sterilized, are common to make the treatment available to the underprivileged, even three dimensional mapping has been extended to study arrhythmias in children by strategic re-use of catheters and electrode patches and sometimes even dividing them to suit the small body size. With the continued follow-up of patients who had undergone device closure or surgery for congenital heart disorders, post procedure tachycardias have to be reckoned with [18]. This is particularly so in institutions were congenital heart disorder corrective surgeries and procedures are regularly undertaken. However very often we are faced with the situation where we have limited options in dealing with a vexing arrhythmia either due to non availability of a drug, limitations imposed by the small size of veins and arteries and the hearts chambers that make interventional electrophysiology in children a very challenging one.

## Drug Therapy of Pediatric Arrhythmias

Despite the better availability of electrophysiological facilities in the country, drug therapy still forms the mainstay of management of arrhythmias in children as is seen the world over. The seminal study conducted by Amit Vora et al [19] although not in the age group for children provided a study in the Indian scenario for widespread use of amiodarone in atrial fibrillation. Acceptability for other arrhythmias in this population soon followed and today amiodarone is in wide use. Caution has to be exercised in careful follow up for its thyroid and eye adverse reactions. Non availability in India of flecainide (in some cities), propafenone, quinidine, disopyramide, ibutilide etc has led to this unavoidable fondness for amiodarone.

## Resources for Pediatric Electrophysiology in India

India has reportedly only 14 centers where Pediatric cardiac care and expert surgical care can be offered to neonates and infants and there are only 25 pediatric cardiologists and a dozen pediatric cardiac surgeons - a small fraction of what is actually required [20]. Such being the state of Pediatric cardiology, the status of pediatric electrophysiology as a discipline is almost non-existent.

The Pediatric Cardiac Society of India has been conducting a session on Pediatric Electrophysiology at its annual meetings which are of great value. We had organized a pediatric electrophysiology symposium from 4th to 6th January, 2008 at Chennai with support from the Indian Heart Rhythm Society and the Cardiological Society of India called the TCPES2008. This was the second of this type with the first one conducted at the Railway Hospital, Perambur, Chennai in January 2002. Its aim was to provide a forum where the unique problems of managing arrhythmias in children could be thrashed about and a consensus on management approached. This has been possible by the participation of an erudite group of pediatric electrophysiologists from around the world. The deliberations of this meeting have been brought out as a supplement of the Indian Pacing and Electrophysiology Journal, the official journal of the Indian Heart Rhythm Society. The articles in this supplement will give state-of-the-art information on paediatric cardiac electrophysiology to the cardiovascular professionals. We hope that this supplement will be of great use to cardiology and electrophysiology fellows who wish to take up their career in paediatric cardiac electrophysiology.

## Figures and Tables

**Figure 1A:**
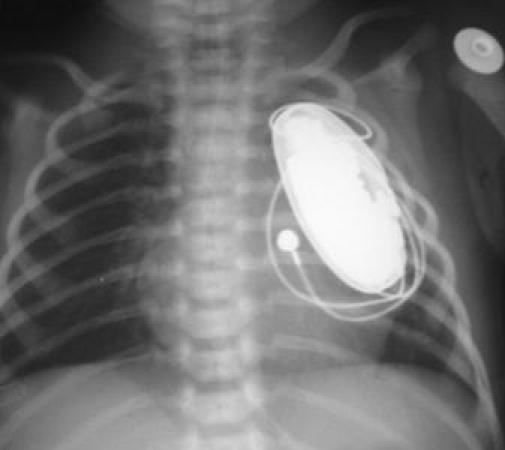
Chest roentgenogram shows the generator in extrapleural location in the infant. Reprinted with permission from Agarwal R, et al Extrapleural intrathoracic implantation of permanent pacemaker in the pediatric age group. Ann Thorac Surg. 2007;83:1549-52. Elsevier © 2007, The Society of Thoracic Surgeons.

**Figure 1B:**
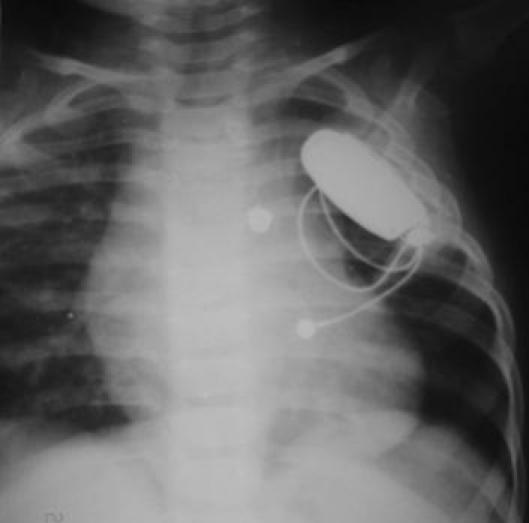
Follow-up roentgenogram in the same child shows normal lung growth at 20 months. Reprinted with permission from Agarwal R, et al Extrapleural intrathoracic implantation of permanent pacemaker in the pediatric age group. Ann Thorac Surg. 2007;83:1549-52. Elsevier © 2007, The Society of Thoracic Surgeons.

**Figure 2A:**
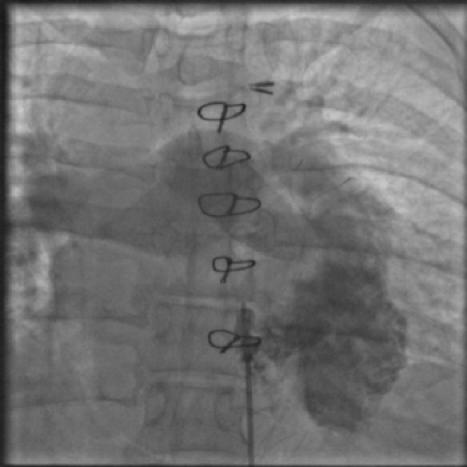
Right ventricular angiography showing a left sided right atrium and ventricle in a girl operated for a large VSD and corrected transposition who was having hemodynamically unstable VT.

**Figure 2B:**
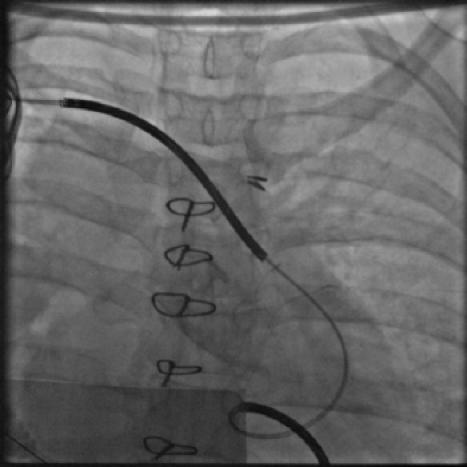
Automatic implantable defibrillator implanted.

**Figure 3A:**
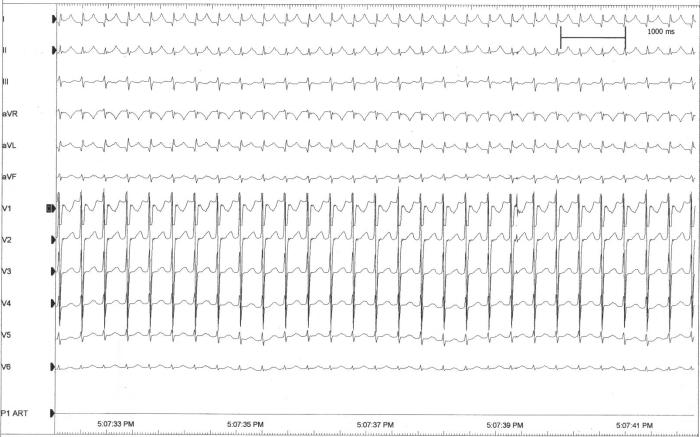
ECG of a narrow QRS incessant tachycardia unresponsive to propranolol, digoxin, amiodarone and flecainide that caused congestive heart failure and low output state leading to ventilation and anuria in a twenty seven month old child with dextrocardia and a left superior vena cava.

**Figure 3B:**
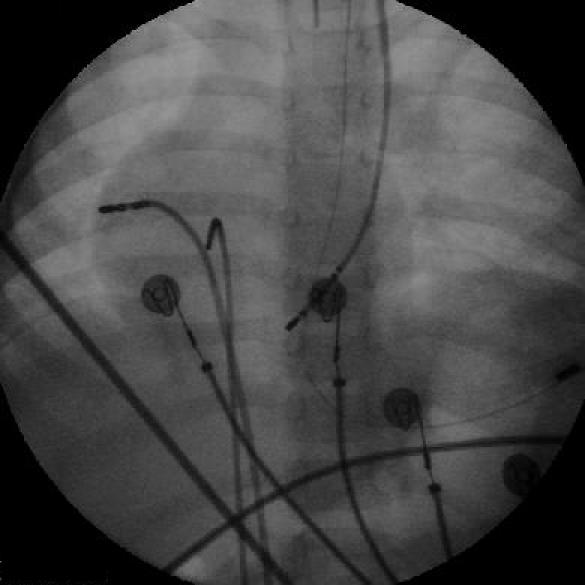
Cine showing the radiofrequency ablation catheter in the right free wall position, a His catheter and a coronary sinus catheter introduced through a left superior vena cava.

**Figure 3C:**
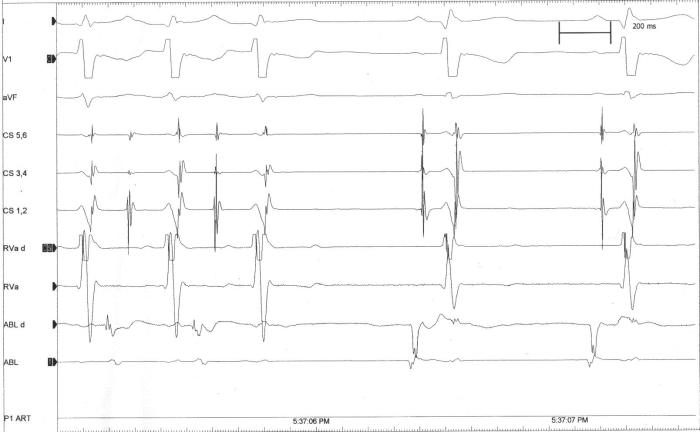
Intracardiac during successful radiofrequency ablation of a right free wall accessory pathway terminating the tachycardia.
